# Hypomethylated *Fgf3* is a potential biomarker for early detection of oral cancer in mice treated with the tobacco carcinogen dibenzo[*def*,*p*]chrysene

**DOI:** 10.1371/journal.pone.0186873

**Published:** 2017-10-26

**Authors:** Yuan-Wan Sun, Kun-Ming Chen, Yuka Imamura Kawasawa, Anna C. Salzberg, Timothy K. Cooper, Carla Caruso, Cesar Aliaga, Junjia Zhu, Krishne Gowda, Shantu Amin, Karam El-Bayoumy

**Affiliations:** 1 Department of Biochemistry & Molecular Biology, Pennsylvania State University College of Medicine, Hershey, PA, United States of America; 2 Department of Pharmacology, Pennsylvania State University College of Medicine, Hershey, PA, United States of America; 3 Institute for Personalized Medicine, Pennsylvania State University College of Medicine, Hershey, PA, United States of America; 4 Department of Comparative Medicine, Pennsylvania State University College of Medicine, Hershey, PA, United States of America; 5 Department of Pathology, Pennsylvania State University College of Medicine, Hershey, PA, United States of America; 6 Department of Public Health Sciences, Pennsylvania State University College of Medicine, Hershey, PA, United States of America; Centre de Recherche en Cancerologie de Lyon, FRANCE

## Abstract

Genetic and epigenetic alterations observed at end stage OSCC formation could be considered as a consequence of cancer development and thus changes in normal or premalignant tissues which had been exposed to oral carcinogens such as Dibenzo[*def*,*p*]chrysene (DBP) may better serve as predictive biomarkers of disease development. Many types of DNA damage can induce epigenetic changes which can occur early and in the absence of evident morphological abnormalities. Therefore we used ERRBS to generate genome-scale, single-base resolution DNA methylomes from histologically normal oral tissues of mice treated with DBP under experimental conditions known to induce maximum DNA damage which is essential for the development of OSCC induced by DBP in mice. After genome-wide correction, 30 and 48 differentially methylated sites (DMS) were identified between vehicle control and DBP treated mice using 25% and 10% differences in methylation, respectively. RT-PCR was further performed to examine the expressions of nine selected genes. Among them, *Fgf3*, a gene frequently amplified in head and neck cancer, showed most prominent and significant gene expression change (2.4× increases), despite the hypomethylation of *Fgf3* was identified at >10kb upstream of transcription start site. No difference was observed in protein expression between normal oral tissues treated with DBP or vehicle as examined by immunohistochemistry. Collectively, our results indicate that *Fgf3* hypomethylation and gene overexpression, but not protein expression, occurred in the early stage of oral carcinogenesis induced by DBP. Thus, *Fgf3* hypomethylation may serve as a potential biomarker for early detection of OSCC.

## Introduction

Head and neck cancer (HNC) is the sixth most common malignancy worldwide [1]. Approximately 48% of HNC cases occur in the oral cavity, of which 90% are oral squamous cell carcinoma (OSCC) [2]. The development of OSCC involves multiple steps from hyperplastic lesion, through dysplasia and carcinoma *in situ* to invasive disease [3]. This process is a result of multiple accumulated genetic and epigenetic changes in a variety of cellular pathways [2]. Despite advances in treatment modalities, 5-year survival of OSCC has remained at ~50% for the past decades, mostly due to the high risk of developing secondary primary tumors. Early detection of OSCC represents one of the most promising approaches to improving survival [4].

Animal models that closely recapitulate the molecular and pathological process of oral carcinogenesis can assist in the identification of molecular targets for early detection and in monitoring the efficacies of therapeutic and chemopreventive agents [2]. We previously reported that topical application of dibenzo[*def*,*p*]chrysene (also known as dibenzo[*a*,*l*]pyrene, DBP) into the oral cavity induced OSCC in B6C3F_1_ mice [5]. We recently reviewed the literature and concluded that DBP is the most potent carcinogenic polycyclic aromatic hydrocarbons (PAH) found in tobacco smoke to induce oral cancer in animal models [6]. Tobacco consumption is a major risk factor of oral cancer, and PAH, one of the major classes of carcinogens in tobacco smoke, have been recognized as potential etiological agents for oral cancer [7–10]. Although DBP is found at lower levels in environmental sources and in cigarette smoke than the most extensively studied prototype PAH benzo[*a*]pyrene (BaP), its remarkable carcinogenicity in animal models suggests it can pose a cancer risk to humans [11].

Similar to other PAH carcinogens, DBP can be metabolically activated to yield various types of reactive metabolites including diol-epoxides, *o*-quinones, and radical cations. It is expected that these metabolites could damage DNA through formation of covalent DBP-DNA adducts, generation of reactive oxygen species (ROS) and depurinating adducts [11, 12]. Many types of DNA damage (covalent DNA adducts, oxidative lesions, abasic sites, photodimers, etc.) have been shown to alter DNA methylation *via* various mechanisms associated with the formation of DNA lesions or by inhibition of DNA methyltransferases (*Dnmts*) [13–16]. DNA methylation is an epigenetic mechanism for regulating gene function that is usually associated with inhibition of promoter activity and chromatin repression. In the early and precancerous stages, DNA methylation patterns display specific aberrations and may confer susceptibility to further genetic or epigenetic changes [17]. Hypermethylation (gain of methylation) of tumor suppressor genes as well as hypomethylation (loss of methylation) of oncogenes are common events in carcinogenesis. Aberrant DNA methylation has been identified in the progression of OSCC and been implicated as key events in oral carcinogenesis [17–20]. With the advent of genome-wide technology in recent years, new hypermetylated as well as hypomethylated loci were also discovered in different stages of oral cancer [21–23]. Molecular alterations that occur in OSCC could represent the consequence of cancer development and thus alterations in histologically normal tissues and persist in OSCC could potentially serve as early biomarkers for disease progression. Furthermore, the use of DNA methylation as markers for early detection of cancer may provide better sensitivity and more stable samples as compared to the use of protein or mRNA [24].

In this study, we examined the effect of DBP on DNA methylation in histologically normal oral tissues of mice treated with DBP. Based on our previously established animal bioassays [5, 25], oral tissues with maximum levels of DBP-DNA adducts were isolated from the same anatomic sites of mice treated with DBP or vehicle and subjected to genome-wide DNA methylation analysis using Enhanced Reduced Representation Bisulfite Sequencing (ERRBS) method coupled with next generation sequencing [26].

## Materials and methods

### Animals and carcinogen treatment

To mimic the bioassay employed in our previous carcinogenicity and DNA adduct studies [5, 25, 27, 28], eight week-old female B6C3F1/J mice (Jackson Laboratories, Bar Harbor, ME) were used in this study. Mice were quarantined for about 1 week before treatment. All mice were kept on a 12-hr light:12-hr dark cycle, maintained at 50% relative humidity and 21± 2°C, and were fed with AIN-93M diet (5% corn oil), and water *ad libitum*. The bioassay was carried out in accordance with the NIH Guide for the Care and Use of Laboratory Animals and was approved by Institutional Animal Care and Use Committee.

Mice received topical treatment of DBP (24 nmol, 3 times per week for 5 weeks) in the oral cavity and were euthanized 48 h after the last dose; this time point was selected based on our previous study showing maximum levels of DNA adducts [25]. Animals treated with dimethyl sulfoxide (DMSO) as the vehicle were used as control. At termination, mice were euthanized by CO_2_ asphyxiation; oral tissues were isolated from the same anatomic sites of mice treated with vehicle or DBP (soft tissues of the oral cavity, including the buccal mucosa and floor of the mouth as well as soft tissues attached to the hard palate) and pooled together for DNA extraction.

### Whole genome ERRBS analysis

Genomic DNA from oral tissues of mice treated with DBP or DMSO were extracted and purified according to the DNeasy Blood and Tissue kit (Qiagen). DNA was then subjected to ERRBS to examine the DNA methylation status in CpG sites and the surrounding regions with single nucleotide resolution [26]. Briefly, 50 ng of genomic DNA was digested by MspI (Thermo Scientific), which recognizes and cleaves C^CGG sites, followed by end repair, adenylation and adapter ligation using NEXTflex Bisulfite-Seq Library Prep Kit and NEXTflex Bisulfite-Seq Barcodes (Bioo Scientific) with a modification of bead size selection to capture MspI fragments of 70–320 bp size. The resulting libraries were bisulfite-converted by using EZ DNA Methylation Kit (Zymo Research), followed by 18 cycles of PCR amplification using the NEXTflex Bisulfite-Seq U+PCR Master Mix and NEXTflex Primer Mix (BioO Scientific). Purified and quantified libraries were pooled at 6 samples per sequencing lane and read by 1X50 bp on HiSeq 2500 (Illumina). CASAVA-demultiplexed.fastq files were subjected to downstream analyses.

### Sequence alignments and differential methylation analysis

Base calls of bisulfite treated sequencing reads with phred quality scores < 20 were trimmed and the adaptor was cut using trim_galore v0.3.3 (Babraham Bioinformatcis, UK). Resulting reads were mapped to the mm9 mouse assembly and methylation calls were performed using Bismark v0.10.1 (Babraham Bioinformatcis, UK). The alignment workflow, which utilized Bismark [29] started by converting sequencing reads *in silico* into a fully bisulfite-converted form [C-to-T or G-to-A version (equivalent to a C-to-T conversion on the reverse strand)]. Then, each of them was aligned to equivalently converted versions of the reference genome using two parallel instances of the short read aligner Bowtie [29]. The methylation state of positions involving cytosine is determined by comparing the read sequence with the corresponding genomic sequence. The methylKit (version 0.9.2) R package [30] was then used to calculate the differential methylation between control *vs* DBP using the following parameters: bases with coverage below 10× and bases that had more than 99.9^th^ percentile of coverage were discarded in each sample, read coverage distributions between samples were normalized and reads on both strands of a CpG dinucleotide were merged to provide better coverage. This software implements the Benjamini-Hochberg false discovery (FDR)-based method for *P*-value correlation and only differentially methylated bases with *q*-value < 0.01 and percent methylation difference > 25% and >10%were extracted. These were annotated with genetic parts information from the UCSC Table Browser (mm9 refGene table).

### Quantitative real-time polymerase chain reaction (RT-PCR)

Total RNA was extracted and purified from oral tissues of mice treated with DBP or vehicle (*n* ≥ 3 per group) according to the RNeasy kit (Qiagen Inc. Hilden, Germany). Total RNA was reverse transcribed in the presence of SuperScript II reverse transcriptase (Invitrogen Inc. Carlsbad, CA). Real-time PCR was performed using TaqMan® primer/probe sets on a QuantStudio™ 12K Flex Real-Time PCR System (Life Technologies,Carlsbad, CA). The primer assay IDs are listed in S1 Table. Relative gene expression was assessed using TATA-binding protein (TBP) or β-actin as internal reference genes. All reactions were performed in triplicate and fold changes were determined using the 2 ^-ΔΔCt^ method. The C_t_ is the value where the real-time PCR curve crosses the threshold in the linear part of the curve [31].

### Histology and immunohistochemistry

Normal oral tissues harvested from mice treated with DBP and DMSO for 5 weeks were fixed in formalin, embedded in paraffin and then sectioned. Hematoxylin and eosin (H&E) staining was conducted (*n* = 3 per group) to examine the histological status of oral tissues. Normal oral tissues from mice treated with DBP for 38 weeks as well as OSCC induced by DBP were obtained from our previous studies [5] and were processed as described above.

Immunohistochemistry for FGF3 in normal oral tissues and archived OSCC (obtained from our previous bioassay [5] were performed using an indirect immunoperoxidase method in an automated Ventana Discovery XT stainer. Briefly, 5 μm sections were heated to 60°C, deparaffinized in xylene, rehydrated in graded alcohols, and rinsed with water. The tissue sections were then subjected to a standard microwave antigen retrieval procedure before being blocked by hydrogen peroxide. Slides were incubated sequentially with diluted anti-FGF3 polyclonal antibodies (Novus Biologicals Inc, cat#NB200-603) overnight at 4°C and then with biotinylated goat anti-rabbit secondary antibodies (1:1200 dilution, Vector Laboratories, Inc) for 40 min at room temperature. After washing, sections were incubated at streptavidin peroxidase (BoehringerMannheim, Indianapolis, IN) and visualized by incubating with 3, 3-diaminobenzidine-tetrahydrochloride solution (DAKO Corporation, Carpinteria, CA) for 1 min. The slides were thoroughly washed with tap water and counterstained with a modified Harris hematoxylin (Fisher Scientific, Fairlawn, NJ). All slides were examined by ACVP diplomate veterinary pathologist (TK) blinded to treatment. Additional examination was performed by another pathologist (CC). All light microscopic images were obtained with an Olympus BX51 microscope and DP71 digital camera using cellSens Standard 1.12 imaging software (Olympus America, Center Valley, PA).

## Results

### Genome-wide bisulfite sequencing in oral tissues of mice

DNA isolated from normal oral tissues of mice treated with DBP or DMSO for 5 weeks was subjected to ERRBS analysis which is a modified version of RRBS with improved coverage of regions both within and outside CpG islands. Combined with next generation sequencing, this approach provides a quantitative, single nucleotide resolution on the status of DNA methylation in CpG sites as well as the surrounding regions [26]. The sequencing data have been deposited in the National Center for Biotechnology Information Gene Expression Omnibus database (Accession No: GSE89916).

The coverage of each control (DMSO) and treated (DBP) sample are illustrated in S1 Fig which the read depths are displayed on the x-axis and the total numbers of reference bases that occupy each read depth are displayed on the y-axis. Consistent with the literature [32], the overall methylation levels of CpG showed a similar bimodal distribution among samples (S2 Fig). Summary of ERRBS performance for individual samples is shown in S2 Table. An average of 3.5 million Illumina sequencing reads were generated per sample; of these, 73% were mapped to either strand of the mouse genome (mm9). ERRBS covered an average of 31 million cytosines and 2.3 million CpGs per sample. It is noted that the average percentage of cytosine methylated in CpG context is lower in the DBP treated group than that in the control group (35.2% *vs*. 40.7%), indicating an overall hypomethylation effect induced by DBP (S2 Table). The overall distribution of sequencing read at gene promoters and annotated CpG islands are shown in S3 Fig. The percentage of promoters covered with sequencing depth ≥ 10 × is 57% and the percentage of CpG islands 90%.

### Effect of DBP treatment on DNA methylation

**Fig 1:**
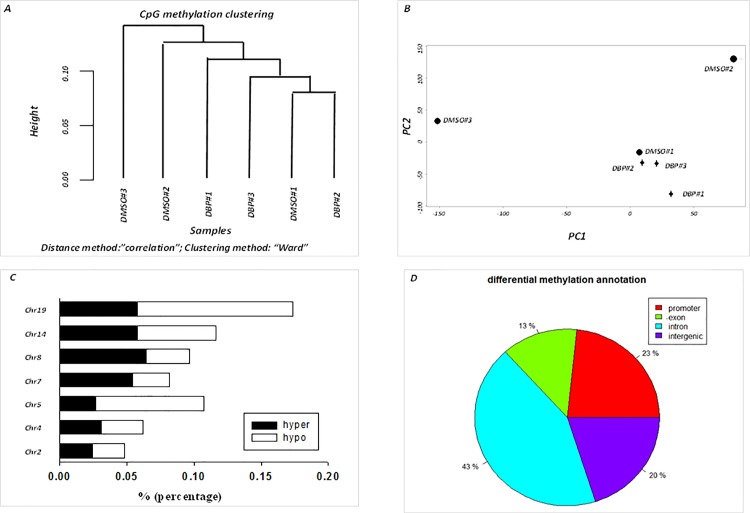
Analysis of methylation data obtained by ERRBS in oral tissues of mice treated with DBP or DMSO. (A) Unsupervised analysis of DNA methylation using hierarchical clustering. Distance  =  1-Pearson correlation, Ward's agglomeration method. Cluster: filtered (count > = 10, coverage < = 99.9^th^ percentile), normalized, destranded. (B) Principal component analysis (PCA) of methylated data. (C) Percentage of hyper- and hypo-methylated sites out of all covered CpGs per chromosomes (Chr). Solid bar represents proportion of hypermethylated sites and open bar represents hypomethylated ones. Filtered, normalized, destranded, *q* < 0.01, % methylation difference > 25%. (D) Annotation of differential methylation distribution.

Comparative methylation analysis was conducted between DBP and DMSO-treated groups to identify global DNA methylation differences using MethylKit (version 0.9.2) R package [30]. Function “normalizeCoverage” was applied which normalizes read coverages between samples to avoid bias introduced by systematically more sequenced samples. Our results showed that there was a high degree of correlation among all of the samples as the pair-wise Pearson correlation coefficients ranged from 0.95–0.97 (data not shown). Both unsupervised analyses of hierarchical clustering (1-Pearson correlation distance + Ward clustering method) (Fig 1A) and principal component analysis (PCA) revealed a tendency of clustering in DBP-treated samples but much less tendency in control samples (Fig 1B). Stacking bar plots in Fig 1C show the percentages of hypermethylated and hypomethylated sites out of all covered CpGs for each chromosome (Chr). Solid bar represents proportion of hypermethylation and open bar represents hypomethylation relative to control (DMSO-treated group). Chr19 has the highest percentage of combined hyper- and hypo-methylated region per chromosome, followed by Chr14, Chr5, Chr8, Chr7, Chr4 and Chr2. Only CpGs with *q*-value <0.01 and methylation difference ≥ 25% are shown. About 23% of DMS are located in promoters, 13% in exons, 43% in introns and 20% in intergenic regions (Fig 1D).

The methylation analysis was performed using strict parameters (*q*-value < 0.01, 25% methylation difference, minimum depth of 10) and the default “unite” function in the methylKit® packaget, which requires every sample in the comparison to have the methylation site call. With these parameters, only 30 differential methylation sites were identified as shown in Table 1 (full table of genomic results was included in S3 Table). Thus, we relaxed the 25% methylation difference to 10% and 48 differential methylation sites were identified (S4 Table). DMS were mapped to genes including pyruvate kinase, muscle (*Pkm*), β-catenin (*Ctnnb1*), and fibroblast growth factor 3 (*Fgf3*) that are known to be involved in tumor development including OSCC.

**Table 1:** List of differentially methylated sites identified in histologically normal oral tissues of mice treated with DBP (methylation difference > 25%).

Feature name	Gene	Methylation Difference*	Prom	Exon	Intron	Distance to Feature
NM_028417	Ttc9b	47.82836211	0	0	1	3885
NM_011099	Pkm	43.38028169	1	0	1	396
NM_001010836	Ppp1r13l	43.26828307	0	1	0	8827
NM_009498	Vamp3	38.83295195	0	0	1	-71795
NM_174854	Disc1	36.25	0	0	1	158596
NM_174854	Disc1	35.31559729	0	0	1	158606
NM_026796	Smyd2	32.76743337	0	1	0	48511
NM_001165902	Ctnnb1	31.54929577	1	0	1	913
NM_001081278	Tbc1d4	31.30134429	1	1	0	65
NM_026675	Nudt22	29.09571077	1	1	0	729
NM_001145857	Lrp4	25.92592593	1	0	0	-378
NM_017467	Zfp316	25.29163315	0	0	0	-17980
NR_040542	1700036G14Rik	-26.90181336	0	0	0	-130664
NM_001195268	Cbarp	-27.81201849	0	1	0	5372
NR_040704	4930563F08Rik	-28.34737828	0	0	1	-162625
NM_201361	Rmdn2	-28.51253865	0	0	1	48092
NM_001164352	Efemp2	-28.94927536	1	0	1	582
NM_015748	Slit1	-30.37280702	0	0	1	67038
NM_030022	Grifin	-30.91515531	0	0	0	15755
NM_001101546	Tmem233	-31.0515873	0	0	1	-84149
NR_035421	Mir1190	-31.69848585	0	0	1	39110
NM_008721	Npdc1	-32.82083139	0	1	0	-3532
NM_028658	Ppp1r21	-33.67514356	0	0	1	23064
NM_198423	Bahcc1	-34.74658869	0	0	0	-11592
NM_001033267	Qrich2	-36.78972713	0	0	0	-2322
NM_177150	Cenpt	-37.08920188	0	0	1	1277
NM_008007	Fgf3	-37.63736264	0	0	0	-10072
NM_183175	C1qtnf9	-39.23549718	0	0	1	-44173
NM_028658	Ppp1r21	-42.79624893	0	0	1	23042
NM_013848	Ermap	-43.33333333	1	0	0	-839

*(+): hypermethylation; (-): hypomethylation

### Correlation between DNA methylation and gene expression

**Fig 2:**
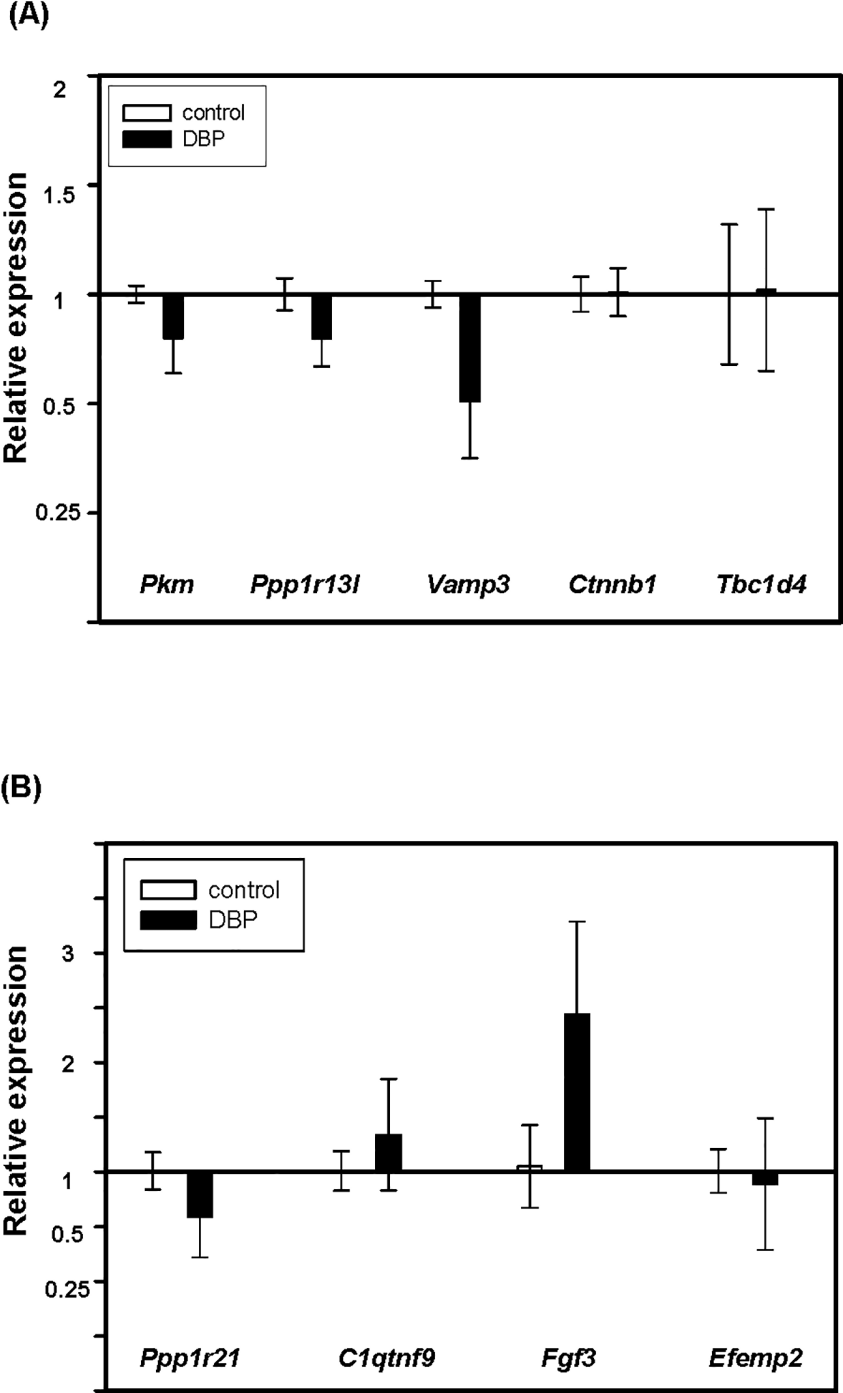
Relative gene expressions by quantitative real-time PCR in oral tissues of mice treated with DBP or DMSO. The relative expressions of (A) hypermethylated genes (*Pkm*, *Ppp1r13I*, *Vamp3*, *Ctnnb1 and Tbc1d4*) and (B) hypomethylated genes (*Ppp1r21*, *C1qtnf9*, *Fgf3*, *Efemp2*) identified in ERRBS were determined using the 2 ^-ΔΔCt^ method. Bars represent the mean normalized expression (±SD) of genes in DBP-treated mice relative to control. Data were normalized using endogenous housekeeping genes as the reference and untreated control as the calibrator (with expression equal to 1).

The impact of DNA methylation changes on gene expression was determined by RT-PCR using TaqMan® primer/probe sets. The hypermethylated genes *Pkm*, *Ppp1r13l* [protein phosphatase 1, regulatory (inhibitor) subunit 13 like], *Vamp3 (*vesicle-associated membrane protein 3), *Ctnnb1*, and *Tbc1d4* (BC1 domain family, member 4) and hypomethylated genes *Ppp1r21* (protein phosphatase 1, regulatory subunit 21), *C1qtnf9* (C1q and tumor necrosis factor related protein 9), *Fgf3* and *Efemp2* (epidermal growth factor-containing fibulin-like extracellular matrix protein 2) were selected. These genes are either known components of the signaling cascades linked to the top canonical pathways (*Ctnnb1*, *Vamp3*, *Fgf3 and Pkm*) or are potentially relevant for establishment of malignant transformation. Fig 2A and 2B show the relative expressions of selected hypermethylated and hypomethylated genes in oral tissues treated with DBP *vs* DMSO, respectively (*n* ≥ 3 per group). Among them, gene expression changes of *Fgf3* (2.4×), *Vamp3* (0.49×) and *Ppp1r21* (0.51×) were most prominent; *Pkm* (0.80×), *Ppp1r13I* (0.80×), *Efemp2* (0.89×) and *C1qtnf9* (1.3×) were less prominent; and no changes were observed in *Ctnnb1* and *Tbc1d4*. The changes of gene expression levels of *Fgf3* and *Vamp3* are consistent with their alterations in DNA methylation: single base hypomethylation (152014445, methylation differences = -37.63, intergenic region) and hypermethylation (150503856, methylation differences = 38.83, intron region), respectively. It was also noted that *Ppp1r21* was hypomethylated but gene expression was decreased.

Since aberrant DNA methyltransferases (*Dnmts*) activity has been reported in numerous cancers [33–35], we examined the expression of the 3 major *Dnmts*: *Dnmt1*, *Dnmt3a and Dnmt3b* which are known to be involved in DNA methylation maintenance (*Dnmt1*) or *de novo* methylation (*Dnmt3a* and *Dnmt3b*). However, no significant gene expression changes were observed between DBP and vehicle treatments (data not shown).

### Immunohistochemistry of FGF3 in normal oral tissues and OSCC

**Fig 3:**
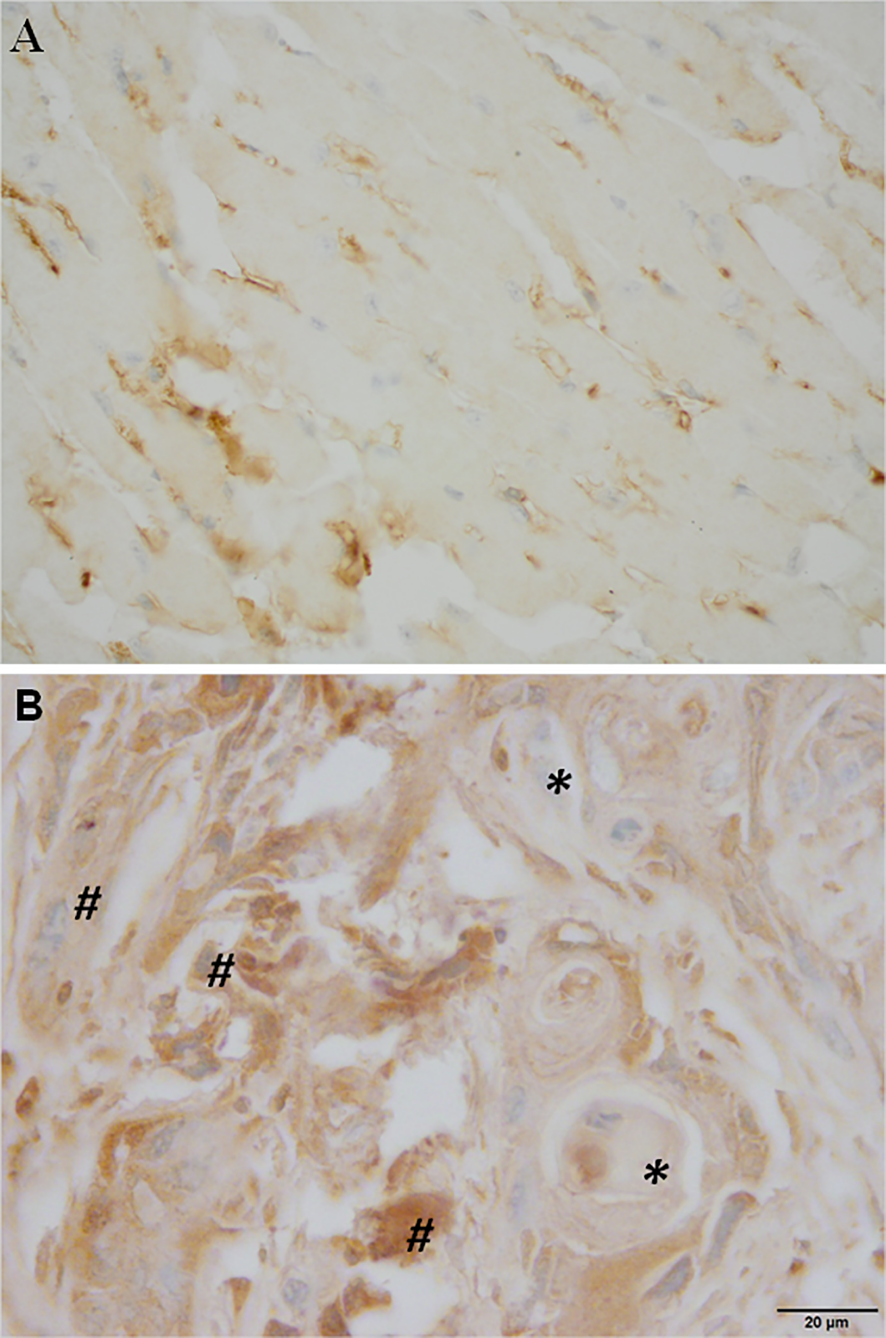
**Immunohistochemical reactivity for FGF3 in (A) normal oral tissues and (B) oral squamous cell carcinoma (OSCC) obtained from mice treated with DBP**. A: normal oral tissues showing no positive immunostaining in epithelial cells but diffuse positive cytoplasmic staining in interstitial macrophages and fibroblasts (600×). B: OSCC induced by DBP exhibiting positive FGF3 immunostaining in tumor cells (marked with asterisk*) and stroma (marked with #) (600×).

*Fgf3* has the most prominent gene expression changes among all the genes examined by RT-PCR and has been reported to be amplified in head and neck cancers [36–38]. To examine if hypomethylation and gene amplification of *Fgf3* will also result in changes in protein expression, FGF3 expression was examined by immunohistochemistry. Both oral tissues from mice treated with DBP or DMSO for 5 weeks (*n* ≥ 3 per group) were histologically normal as determined by H&E staining (data not shown). We found that FGF3 immunostaining was negative in all of the normal oral epithelial cells, regardless of DBP or DMSO treatments or duration of treatments (5 or 38 weeks) (Fig 3A). When comparing FGF3 immunostaining in the stroma of normal oral tissues by semiquantitative H-score (staining intensity × percentage of positive cells), no significant differences were noted between DBP and DMSO treatments at either 5 or 38 weeks (*p* = 0.612 and 0.555, respectively, *n* ≥ 4, two tailed *t-test*). However, we found that H-scores obtained at 38 weeks were significantly higher than those obtained from 5 weeks in both DBP and DMSO-treated groups (*p* = 0.002 and 0.009, respectively; *n* ≥ 4, two tailed *t-test)*. These results indicated that increases of FGF3 expression in the stroma of normal oral tissues appeared to be time dependent.

We further examined the expression of FGF3 in OSCC induced by DBP after 38 weeks of treatment; these tumors were obtained from our previous study [5]. We found that 5 of 5 animals (100%) with OSCC showed positive FGF3 immunostaining (Fig 3B); among 8 OSCC, 5 show positive staining (62.5%). The expression of FGF3 protein in the tumor cells but not in the normal oral epithelium cells suggested that *Fgf3* hypomethylation is an early response to DBP treatment.

## Discussion

Genetic and epigenetic alterations at end stage OSCC formation could be considered a consequence of cancer development and may not provide biomarker for early detection of the disease. Therefore, in the present study, we utilized ERRBS technique to examine genome-scale DNA methylation alterations in histologically normal oral tissue of mice treated topically with DBP under the conditions known to induce the maximum levels of DNA damage in the target organ [27]; such damage was essential to induce OSCC [5, 28]. By querying more than 2 million CpG sites, our study identified 30 and 48 differentially methylated loci using > 25% and 10% differences, respectively. These DMS were mapped to genes including *Fgf3*, *Ctnnb1* and *Pkm*, known to be involved in cancer-related pathways. Our results indicate that DBP can induce aberrant DNA methylation in histological normal oral tissues of mice prior to the detection of OSCC. At the protein level, a positive Fgf3 immunostaining was observed in OSCC-induced by DBP but not in normal oral epithelial cells suggesting that *Fgf3* hypomethylation is an early response to DBP treatment.

It has been reported that cytosine methylation may enhance the formation of DNA adducts at CpG site [14, 39], suggesting that the methylation status can influence the levels of DNA adducts formed which may, in turn, alter DNA methylation. Formation of DNA adducts derived from BaP, the most extensively studied prototype PAH found in tobacco smoke, has been linked to aberrant DNA methylation in several experimental systems [15, 40]. Using methylated CpG island recovery assay (MIRA) combined with microarray analysis, Tommasi et. al, reported the detection of aberrant DNA methylation in seminal vesicles of apparently asymptomatic mice treated with BaP for 6 weeks; moreover, 72% of these aberrant methylation CpG islands coincide with those identified in tumors [41]. However, none of the 30 differentially methylated sites identified in our study coincide with those identified in their study. Although the effects of DNA adducts on DNA methylation may vary depending on the species and types of target organ examined, it is possible that the differences between structures and levels of DNA adducts (or other types of DNA damages) induced by BaP and DBP may alter the patterns and extent of changes in DNA methylation. The ultimate carcinogenic diol-epoxide metabolites of BaP are known to react with DNA to form covalent adducts preferentially at the N2 position of deoxyguanosine (dG), and to a lesser extent, at N6 position of deoxyadenosine (dA); however, our previous studies unequivocally showed preferential DBP adducts formation at dA than dG [27]. Although other types of DNA damage induced by DBP have not been examined in the mouse oral cavity but similar to other PAH carcinogens, DBP can undergo metabolic activation to generate ROS and radical cations which may also contribute to aberrant DNA methylation observed in this study. Moreover, the DNA damage induced by DBP may also cause other epigenetic effects not examined in this study, such as histone modifications, chromatin remodeling and/or microRNA gene modulation, which may trigger the changes in DNA methylation.

Carcinogens may dysregulate DNA methylation machinery *via* alterations of DNA methyltransferases expression or activity. Several lines of evidence have demonstrated that the reactive diol-epoxide metabolites of BaP bind preferentially to methylated CpG sites [14] and inhibit DNA methyltransferases activity, thereby, possibly interrupting the establishment and/or maintenance of DNA methylation patterns [42]. In contrast to BaP, the tobacco-specific carcinogen 4-(methylnitrosamino)-1-(3-pyridyl)-1-butanone (NNK) was found to increase *Dnmt1* expression/activity which was implicated for the hypermethyation (gain of methylation) of several tumor suppressor genes (e.g. *p16*^*ink4A*^ and *Rarβ*) frequently occurred in both NNK-exposed rodent lung tumors and in smokers who developed lung cancer [33, 43]. Under the conditions used in this study, changes observed (fold of changes ≥ 2 or ≤ 0.5) in mRNA expression of *Dnmt1*, *Dnmt3a* and *Dnmt3b* after treatment with DBP for 5 weeks were not significant (data not shown). Whether DBP can alter the activity of DNA methyltransferases remains to be determined.

By real-time qPCR, we further examined the expression of selected genes (*Pkm*, *Ppp1r13l*, *Vamp3*, *Ctnnb1*, *Tbc1d4*, *Ppp1r21*, *C1qtnf9*, *Fgf3* and *Efemp2*) that are known to be involved in the tumor development or potentially relevant for establishment of malignant transformation. We found that *Fgf3* (2.4 ×), *Vamp3* (0.49 ×) and *Ppp1r21* (0.51 ×) showed the most prominent gene expression changes. The functions of *Vamp3* and *Ppp1r21* in carcinogenesis have not been reported and their roles in the development of OSCC need to be explored in future investigations.

*Fgf3* belongs to the large fibroblast growth factor superfamily which consists at least 22 different fibroblasts growth factor genes and possess broad mitogenic cell survival activities and several of them are associated with malignant transformation [44]. Frequent amplification of the *Fgf3* gene has been found in human tumors including head and neck cancer and implicated for neoplastic transformation and tumor progression [36–38]. *Fgfs* signaling are also involved in the regulation of epithelial mesenchymal transition (EMT) pathways [45, 46]. Recent studies suggest the presence of EMT may be a predictor of OSCC progression and prognosis and the expression of mesenchymal genes with tumor progression is often accompanied by an increase in cell motility and the loss of epithelial features [47]. These EMT features are seen not only in cases of OSCC progression, but also oral epithelial dysplasia [48], suggesting EMT changes may be found early in the development of OSCC. Epigenetic alterations can drive the transitions between either ends of EMT spectrum [49]. Thus, modulation of DNA methylation of genes involved in EMT pathway may prevent the progression of tumor development.

## Conclusion

Our study has allowed unbiased assessment of global DNA methylation changes. Moreover, DNA methylation, gene expression and immunohistochemistry of *Fgf3* in a well-defined animal model of OSCC indicated that aberrant DNA methylation of *Fgf3* occurred early in oral carcinogenesis induced by DBP. Amplification of *Fgf3* gene has been found in human head and neck cancer and thus hypomethylation of *Fgf3* may serve as a potential biomarker for early detection of OSCC.

## Supporting information

S1 Table Taqman® Primers used for quantitative real-time PCR. (DOCX)

S2 Table Summary of ERRBS performance. (DOCX)

S3 Table List of full differentially methylated sites identified in histologically normal oral tissues of mice treated with DBP using > 25%methylation difference. (XLSX)

S4 Table List of full differentially methylated sites identified in histologically normal oral tissues of mice treated with DBP using > 10%methylation difference. (XLSX)

S1 Fig Histograms of CpG coverage in oral tissues of mice treated with DMSO (control) or DBP. Top panel: control #1, control#2 and control#3; bottom panel: DBP#1, DBP#2 and DBP #3.
(TIF)

S2 Fig Histograms of percentage of CpG methylation ratio (forward and reverse strand) in oral tissues of mice treated with DBP or DMSO (control). An average of more than 2 million CpG dinucleotides with at least 10× coverage was examined. The overall distributions of methylation level are bimodal.
(TIF)

S3 Fig The overall distribution of sequencing read at gene promoters and annotated CpG island. (TIF)

## References

[1] Torre LA, Bray F, Siegel RL, Ferlay J, Lortet-Tieulent J, Jemal A. Global cancer statistics, 2012. CA: a cancer journal for clinicians. 2015;65(2):87–108. doi: 10.3322/caac.21262 . 25651787 10.3322/caac.21262

[2] Tanaka T, Ishigamori R. Understanding carcinogenesis for fighting oral cancer. Journal of oncology. 2011;2011:603740 doi: 10.1155/2011/603740 ; PubMed Central PMCID: PMC3136173. 21772845 10.1155/2011/603740 PMC3136173

[3] Dionne KR, Warnakulasuriya S, Zain RB, Cheong SC. Potentially malignant disorders of the oral cavity: current practice and future directions in the clinic and laboratory. International journal of cancer. 2015;136(3):503–15. doi: 10.1002/ijc.28754 . 24482244 10.1002/ijc.28754

[4] Pai SI, Westra WH. Molecular pathology of head and neck cancer: implications for diagnosis, prognosis, and treatment. Annual review of pathology. 2009;4:49–70. doi: 10.1146/annurev.pathol.4.110807.092158 ; PubMed Central PMCID: PMC3703474. 18729723 10.1146/annurev.pathol.4.110807.092158 PMC3703474

[5] Guttenplan JB, Kosinska W, Zhao ZL, Chen KM, Aliaga C, DelTondo J, et al Mutagenesis and carcinogenesis induced by dibenzo[a,l]pyrene in the mouse oral cavity: a potential new model for oral cancer. International journal of cancer. 2012;130(12):2783–90. doi: 10.1002/ijc.26344 . 21815141 10.1002/ijc.26344 PMC3596885

[6] El-Bayoumy K, Chen KM, Zhang SM, Sun YW, Amin S, Stoner G, et al Carcinogenesis of the Oral Cavity: Environmental Causes and Potential Prevention by Black Raspberry. Chemical research in toxicology. 2017;30(1):126–44. doi: 10.1021/acs.chemrestox.6b00306 . 28092946 10.1021/acs.chemrestox.6b00306

[7] Radoi L, Luce D. A review of risk factors for oral cavity cancer: the importance of a standardized case definition. Community dentistry and oral epidemiology. 2013;41(2):97–109, e78-91. doi: 10.1111/j.1600-0528.2012.00710.x . 22882534 10.1111/j.1600-0528.2012.00710.x

[8] (Ed.) DKUEO, editor. Overview of Oral Cancer,Oral Cancer, Dr. Kalu U. E. Ogbureke and Christopher Bingham: In Tech; 2012.

[9] Warnakulasuriya S, Sutherland G, Scully C. Tobacco, oral cancer, and treatment of dependence. Oral oncology. 2005;41(3):244–60. doi: 10.1016/j.oraloncology.2004.08.010 . 15743687 10.1016/j.oraloncology.2004.08.010

[10] Schneider K, Roller M, Kalberlah F, Schuhmacher-Wolz U. Cancer risk assessment for oral exposure to PAH mixtures. Journal of applied toxicology: JAT. 2002;22(1):73–83. . 11807932 10.1002/jat.828

[11] Leavitt SA, George MH, Moore T, Ross JA. Mutations induced by benzo[a]pyrene and dibenzo[a,l]pyrene in lacI transgenic B6C3F1 mouse lung result from stable DNA adducts. Mutagenesis. 2008;23(6):445–50. doi: 10.1093/mutage/gen033 . 18573814 10.1093/mutage/gen033

[12] Loeb LA, Harris CC. Advances in chemical carcinogenesis: a historical review and prospective. Cancer research. 2008;68(17):6863–72. doi: 10.1158/0008-5472.CAN-08-2852 ; PubMed Central PMCID: PMC2583449. 18757397 10.1158/0008-5472.CAN-08-2852 PMC2583449

[13] Wachsman JT. DNA methylation and the association between genetic and epigenetic changes: relation to carcinogenesis. Mutation research. 1997;375(1):1–8. . 9129674 10.1016/s0027-5107(97)00003-1

[14] Weisenberger DJ, Romano LJ. Cytosine methylation in a CpG sequence leads to enhanced reactivity with Benzo[a]pyrene diol epoxide that correlates with a conformational change. The Journal of biological chemistry. 1999;274(34):23948–55. . 10446162 10.1074/jbc.274.34.23948

[15] Wilson VL, Jones PA. Inhibition of DNA methylation by chemical carcinogens in vitro. Cell. 1983;32(1):239–46. . 6825170 10.1016/0092-8674(83)90514-7

[16] Wojciechowski MF, Meehan T. Inhibition of DNA methyltransferases in vitro by benzo[a]pyrene diol epoxide-modified substrates. The Journal of biological chemistry. 1984;259(15):9711–6. . 6430903

[17] Demokan S, Dalay N. Role of DNA methylation in head and neck cancer. Clinical epigenetics. 2011;2(2):123–50. doi: 10.1007/s13148-011-0045-3 ; PubMed Central PMCID: PMC3365391. 22704334 10.1007/s13148-011-0045-3 PMC3365391

[18] Weber M, Davies JJ, Wittig D, Oakeley EJ, Haase M, Lam WL, et al Chromosome-wide and promoter-specific analyses identify sites of differential DNA methylation in normal and transformed human cells. Nature genetics. 2005;37(8):853–62. doi: 10.1038/ng1598 . 16007088 10.1038/ng1598

[19] Feinberg AP, Ohlsson R, Henikoff S. The epigenetic progenitor origin of human cancer. Nature reviews. 2006;7(1):21–33. doi: 10.1038/nrg1748 . 16369569 10.1038/nrg1748

[20] Towle R, Garnis C. Methylation-mediated molecular dysregulation in clinical oral malignancy. Journal of oncology. 2012;2012:170172 doi: 10.1155/2012/170172 ; PubMed Central PMCID: PMC3356707. 22645611 10.1155/2012/170172 PMC3356707

[21] Foy JP, Pickering CR, Papadimitrakopoulou VA, Jelinek J, Lin SH, William WN Jr., et al New DNA methylation markers and global DNA hypomethylation are associated with oral cancer development. Cancer prevention research (Philadelphia, Pa. 2015 26342026. 10.1158/1940-6207.CAPR-14-0179 PMC4777304 26342026

[22] Towle R, Truong D, Hogg K, Robinson WP, Poh CF, Garnis C. Global analysis of DNA methylation changes during progression of oral cancer. Oral oncology. 2013;49(11):1033–42. doi: 10.1016/j.oraloncology.2013.08.005 . 24035722 10.1016/j.oraloncology.2013.08.005

[23] Sharma S, Kelly TK, Jones PA. Epigenetics in cancer. Carcinogenesis. 2010;31(1):27–36. doi: 10.1093/carcin/bgp220 ; PubMed Central PMCID: PMC2802667. 19752007 10.1093/carcin/bgp220 PMC2802667

[24] Nagata S, Hamada T, Yamada N, Yokoyama S, Kitamoto S, Kanmura Y, et al Aberrant DNA methylation of tumor-related genes in oral rinse: a noninvasive method for detection of oral squamous cell carcinoma. Cancer. 2012;118(17):4298–308. doi: 10.1002/cncr.27417 . 22252571 10.1002/cncr.27417

[25] Zhang SM, Chen KM, Aliaga C, Sun YW, Lin JM, Sharma AK, et al Identification and quantification of DNA adducts in the oral tissues of mice treated with the environmental carcinogen dibenzo[a,l]pyrene by HPLC-MS/MS. Chemical research in toxicology. 2012;24(8):1297–303. doi: 10.1021/tx200188j . 21736370 10.1021/tx200188j PMC3160270

[26] Akalin A, Garrett-Bakelman FE, Kormaksson M, Busuttil J, Zhang L, Khrebtukova I, et al Base-pair resolution DNA methylation sequencing reveals profoundly divergent epigenetic landscapes in acute myeloid leukemia. PLoS genetics. 2012;8(6):e1002781 doi: 10.1371/journal.pgen.1002781 ; PubMed Central PMCID: PMC3380828. 22737091 10.1371/journal.pgen.1002781 PMC3380828

[27] Zhang SM, Chen KM, Sun YW, Aliaga C, Lin JM, Sharma AK, et al Simultaneous detection of deoxyadenosine and deoxyguanosine adducts in the tongue and other oral tissues of mice treated with Dibenzo[a,l]pyrene. Chemical research in toxicology. 2014;27(7):1199–206. doi: 10.1021/tx5001078 ; PubMed Central PMCID: PMC4106691. 24911113 10.1021/tx5001078 PMC4106691

[28] Chen KM, Guttenplan JB, Zhang SM, Aliaga C, Cooper TK, Sun YW, et al Mechanisms of oral carcinogenesis induced by dibenzo[a,l]pyrene: an environmental pollutant and a tobacco smoke constituent. International journal of cancer. 2013;133(6):1300–9. doi: 10.1002/ijc.28152 . 23483552 10.1002/ijc.28152 PMC3707976

[29] Krueger F, Andrews SR. Bismark: a flexible aligner and methylation caller for Bisulfite-Seq applications. Bioinformatics. 2011;27(11):1571–2. doi: 10.1093/bioinformatics/btr167 ; PubMed Central PMCID: PMC3102221. 21493656 10.1093/bioinformatics/btr167 PMC3102221

[30] Akalin A, Kormaksson M, Li S, Garrett-Bakelman FE, Figueroa ME, Melnick A, et al methylKit: a comprehensive R package for the analysis of genome-wide DNA methylation profiles. Genome biology. 2012;13(10):R87 doi: 10.1186/gb-2012-13-10-r87 ; PubMed Central PMCID: PMC3491415. 23034086 10.1186/gb-2012-13-10-r87 PMC3491415

[31] Schmittgen TD, Livak KJ. Analyzing real-time PCR data by the comparative C(T) method. Nature protocols. 2008;3(6):1101–8. . 18546601 10.1038/nprot.2008.73

[32] Meissner A, Mikkelsen TS, Gu H, Wernig M, Hanna J, Sivachenko A, et al Genome-scale DNA methylation maps of pluripotent and differentiated cells. Nature. 2008;454(7205):766–70. doi: 10.1038/nature07107 ; PubMed Central PMCID: PMC2896277. 18600261 10.1038/nature07107 PMC2896277

[33] Lin RK, Hsieh YS, Lin P, Hsu HS, Chen CY, Tang YA, et al The tobacco-specific carcinogen NNK induces DNA methyltransferase 1 accumulation and tumor suppressor gene hypermethylation in mice and lung cancer patients. The Journal of clinical investigation. 2010;120(2):521–32. doi: 10.1172/JCI40706 ; PubMed Central PMCID: PMC2810088. 20093774 10.1172/JCI40706 PMC2810088

[34] Robertson KD, Uzvolgyi E, Liang G, Talmadge C, Sumegi J, Gonzales FA, et al The human DNA methyltransferases (DNMTs) 1, 3a and 3b: coordinate mRNA expression in normal tissues and overexpression in tumors. Nucleic acids research. 1999;27(11):2291–8. ; PubMed Central PMCID: PMC148793. 10325416 10.1093/nar/27.11.2291 PMC148793

[35] Qu Y, Mu G, Wu Y, Dai X, Zhou F, Xu X, et al Overexpression of DNA methyltransferases 1, 3a, and 3b significantly correlates with retinoblastoma tumorigenesis. American journal of clinical pathology. 2010;134(5):826–34. doi: 10.1309/AJCPHGQ69FXDFWII . 20959668 10.1309/AJCPHGQ69FXDFWII

[36] Somers KD, Cartwright SL, Schechter GL. Amplification of the int-2 gene in human head and neck squamous cell carcinomas. Oncogene. 1990;5(6):915–20. . 2193294

[37] Lese CM, Rossie KM, Appel BN, Reddy JK, Johnson JT, Myers EN, et al Visualization of INT2 and HST1 amplification in oral squamous cell carcinomas. Genes, chromosomes & cancer. 1995;12(4):288–95. . 7539284 10.1002/gcc.2870120409

[38] Worsham MJ, Lu M, Chen KM, Stephen JK, Havard S, Schweitzer VP. Malignant and nonmalignant gene signatures in squamous head and neck cancer. Journal of oncology. 2012;2012:752860 doi: 10.1155/2012/752860 ; PubMed Central PMCID: PMC3335248. 22570652 10.1155/2012/752860 PMC3335248

[39] Parker BS, Cutts SM, Phillips DR. Cytosine methylation enhances mitoxantrone-DNA adduct formation at CpG dinucleotides. The Journal of biological chemistry. 2001;276(19):15953–60. doi: 10.1074/jbc.M009216200 . 11278477 10.1074/jbc.M009216200

[40] Gromova Elizaveta S. OMS, Baskunov Vladimir B., and Geacintov Nicholas E. Impact of Carcinogen-DNA Adducts on DNA Methylation. ACS Symposium Series, Structural Biology of DNA Damage and Repair. 2010; 1041:103–16. doi: 10.1021/bk-2010-1041.ch007

[41] Tommasi S, Zheng A, Yoon JI, Besaratinia A. Epigenetic targeting of the Nanog pathway and signaling networks during chemical carcinogenesis. Carcinogenesis. 2014;35(8):1726–36. doi: 10.1093/carcin/bgu026 . 24480805 10.1093/carcin/bgu026

[42] Lewandowska J, Bartoszek A. DNA methylation in cancer development, diagnosis and therapy—multiple opportunities for genotoxic agents to act as methylome disruptors or remediators. Mutagenesis. 2011;26(4):475–87. doi: 10.1093/mutage/ger019 . 21551264 10.1093/mutage/ger019

[43] Jin H, Chen JX, Wang H, Lu G, Liu A, Li G, et al NNK-induced DNA methyltransferase 1 in lung tumorigenesis in A/J mice and inhibitory effects of (-)-epigallocatechin-3-gallate. Nutrition and cancer. 2015;67(1):167–76. doi: 10.1080/01635581.2015.976314 ; PubMed Central PMCID: PMC4363938. 25437343 10.1080/01635581.2015.976314 PMC4363938

[44] Mizukami T, Togashi Y, Naruki S, Banno E, Terashima M, de Velasco MA, et al Significance of FGF9 gene in resistance to anti-EGFR therapies targeting colorectal cancer: A subset of colorectal cancer patients with FGF9 upregulation may be resistant to anti-EGFR therapies. Molecular carcinogenesis. 2017;56(1):106–17. doi: 10.1002/mc.22476 . 26916220 10.1002/mc.22476

[45] Katoh M, Katoh M. WNT signaling pathway and stem cell signaling network. Clinical cancer research: an official journal of the American Association for Cancer Research. 2007;13(14):4042–5. doi: 10.1158/1078-0432.CCR-06-2316 . 17634527 10.1158/1078-0432.CCR-06-2316

[46] Lamouille S, Xu J, Derynck R. Molecular mechanisms of epithelial-mesenchymal transition. Nature reviews Molecular cell biology. 2014;15(3):178–96. doi: 10.1038/nrm3758 ; PubMed Central PMCID: PMC4240281. 24556840 10.1038/nrm3758 PMC4240281

[47] da Silva SD, Morand GB, Alobaid FA, Hier MP, Mlynarek AM, Alaoui-Jamali MA, et al Epithelial-mesenchymal transition (EMT) markers have prognostic impact in multiple primary oral squamous cell carcinoma. Clinical & experimental metastasis. 2015;32(1):55–63. doi: 10.1007/s10585-014-9690-1 . 25433796 10.1007/s10585-014-9690-1

[48] von Zeidler SV, de Souza Botelho T, Mendonca EF, Batista AC. E-cadherin as a potential biomarker of malignant transformation in oral leukoplakia: a retrospective cohort study. BMC cancer. 2014;14:972 doi: 10.1186/1471-2407-14-972 ; PubMed Central PMCID: PMC4301860. 25518919 10.1186/1471-2407-14-972 PMC4301860

[49] Lindsey S, Langhans SA. Crosstalk of Oncogenic Signaling Pathways during Epithelial-Mesenchymal Transition. Frontiers in oncology. 2014;4:358 doi: 10.3389/fonc.2014.00358 ; PubMed Central PMCID: PMC4263086. 25566498 10.3389/fonc.2014.00358 PMC4263086

