# Supplementary material for: Hypomethylated Fgf3 is a potential biomarker for early detection of oral cancer in mice treated with the tobacco carcinogen dibenzo[def,p]chrysene
Source: PLoS One. 2017 Oct 26;12(10):e0186873. doi: 10.1371/journal.pone.0186873 (PMC5658092; doi:10.1371/journal.pone.0186873)
Supplement: S1 Table — (DOCX) [file pone.0186873.s001.docx]

| **Assay ID** | **Gene symbol** | **Gene title** |
| --- | --- | --- |
| Mm00834102_gH | PKm | pyruvate kinase, muscle |
| Mm01313638_m1 | Ppp1r13l | protein phosphatase 1, regulatory (inhibitor) subunit 13 like |
| Mm01268442_g1 | Vamp3 | vesicle-associated membrane protein 3 |
| Mm00483039_m1 | Ctnnb1 | catenin (cadherin associated protein), beta 1 |
| Mm00557659_m1 | Tbc1d4 | BC1 domain family, member 4 |
| Mm00620000_m1 | Ppp1r21 | protein phosphatase 1, regulatory subunit 21 |
| Mm02392597_m1 | C1qtnf9 | C1q and tumor necrosis factor related protein 9 |
| Mm00433289_m1 | Fgf3 | fibroblast growth factor 3 |
| Mm00445429_m1 | Efemp2 | epidermal growth factor-containing fibulin-like extracellular matrix protein 2 |
