# Supplementary material for: Hypomethylated Fgf3 is a potential biomarker for early detection of oral cancer in mice treated with the tobacco carcinogen dibenzo[def,p]chrysene
Source: PLoS One. 2017 Oct 26;12(10):e0186873. doi: 10.1371/journal.pone.0186873 (PMC5658092; doi:10.1371/journal.pone.0186873)
Supplement: S2 Table — (DOCX) [file pone.0186873.s002.docx]

|  | **Total# of sequencing reads** | **Mapping efficiency(%)** | **Total# of C's analyzed** | **Total**  **Methylated**  **CpG** | **% of C methylated**  **in CpG context** |
| --- | --- | --- | --- | --- | --- |
| **control#1** | 3,728,445 | 73 | 33,882,223 | 2,555,361 | 39.9 |
| **control#2** | 2,856,070 | 74 | 25,748,099 | 2,138,019 | 40.8 |
| **control#3** | 1,857,846 | 73.8 | 16,777,770 | 1,356,740 | 41.3 |
| **DBP#1** | 4,522,925 | 75.7 | 40,350,799 | 2,756,772 | 36.7 |
| **DBP#2** | 3,960,383 | 71.4 | 35,957,141 | 2,693,553 | 34.9 |
| **DBP#3** | 4,071,745 | 70.9 | 36,823,667 | 2,709,740 | 34 |
| **average** | 3,499,569 | 73.1 | 31,589,950 | 2,368,364 | Control:40.7 DBP:35.2 |
