# Supplementary figures and images for: Hypomethylated Fgf3 is a potential biomarker for early detection of oral cancer in mice treated with the tobacco carcinogen dibenzo[def,p]chrysene
Source: PLoS One. 2017 Oct 26;12(10):e0186873. doi: 10.1371/journal.pone.0186873 (PMC5658092; doi:10.1371/journal.pone.0186873)

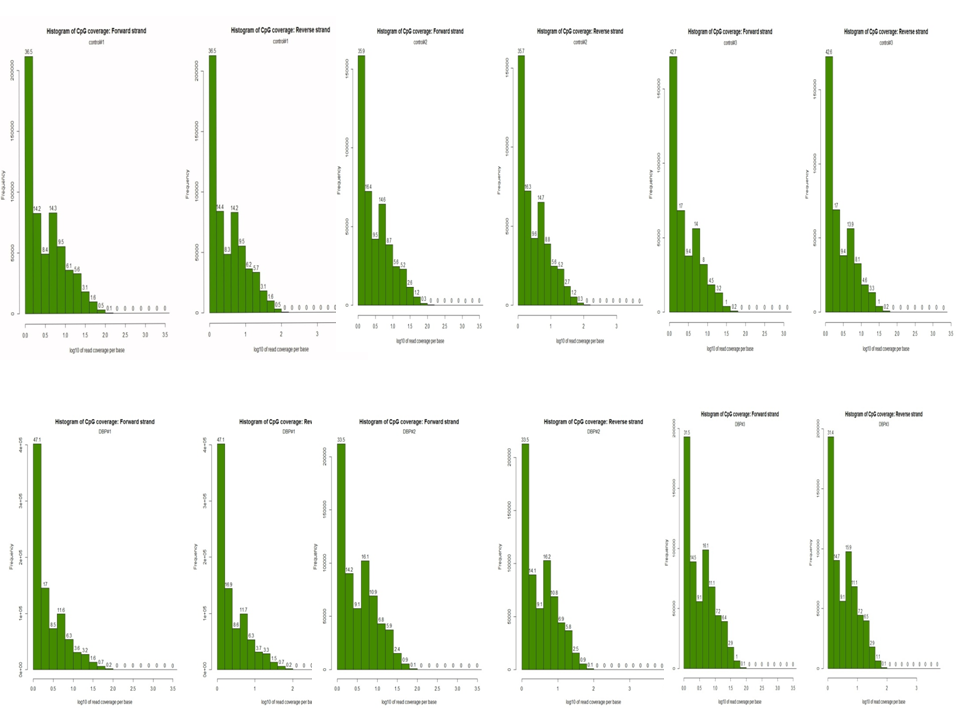

Supplement: S1 Fig — Top panel: control #1, control#2 and control#3; bottom panel: DBP#1, DBP#2 and DBP #3. (TIF) [file pone.0186873.s005.tif]

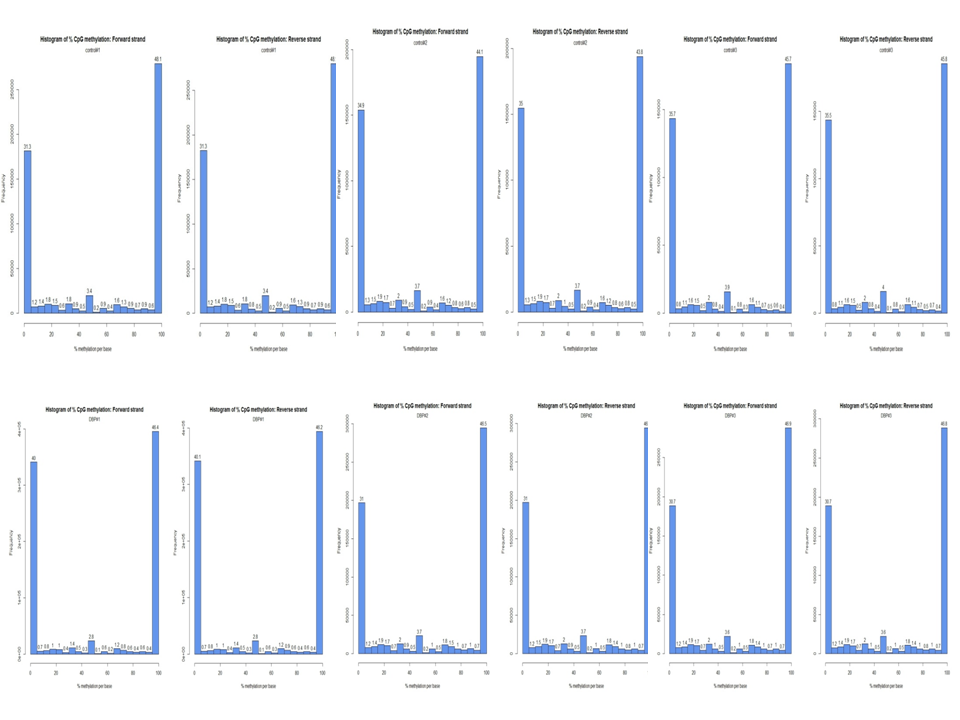

Supplement: S2 Fig — An average of more than 2 million CpG dinucleotides with at least 10× coverage was examined. The overall distributions of methylation level are bimodal. (TIF) [file pone.0186873.s006.tif]

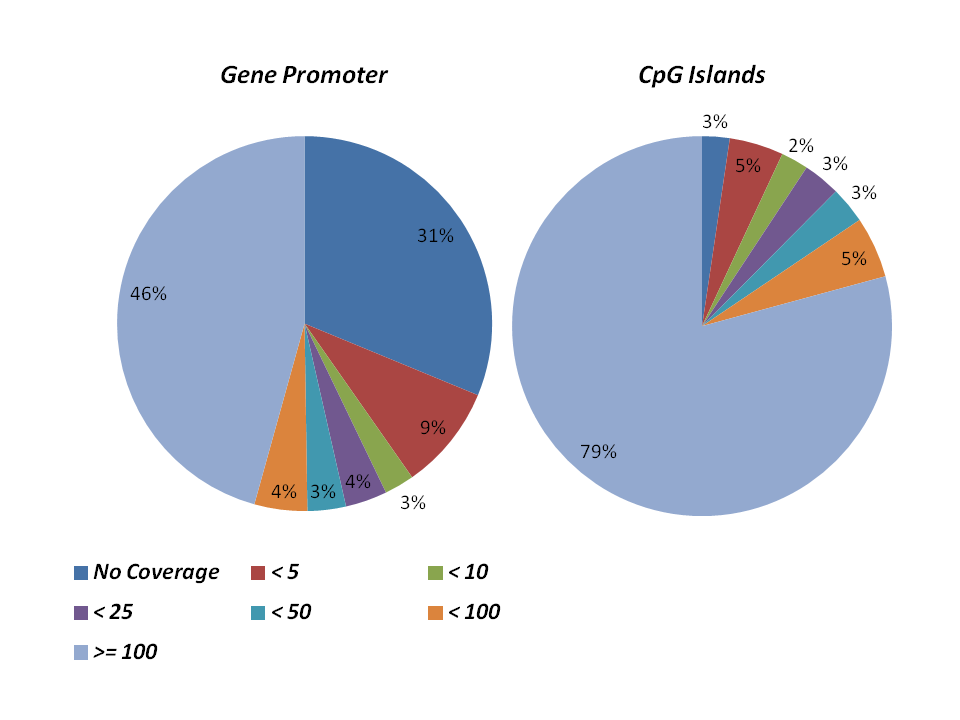

Supplement: S3 Fig — (TIF) [file pone.0186873.s007.tif]
